# The first crystal structure of CD8αα from a cartilaginous fish

**DOI:** 10.3389/fimmu.2023.1156219

**Published:** 2023-04-14

**Authors:** Zhao Jia, Jianhua Feng, Helen Dooley, Jun Zou, Junya Wang

**Affiliations:** ^1^ Key Laboratory of Exploration and Utilization of Aquatic Genetic Resources, Ministry of Education, Shanghai Ocean University, Shanghai, China; ^2^ International Research Center for Marine Biosciences, Ministry of Science and Technology, Shanghai Ocean University, Shanghai, China; ^3^ National Demonstration Center for Experimental Fisheries Science Education, Shanghai Ocean University, Shanghai, China; ^4^ Department of Microbiology and Immunology, University of Maryland School of Medicine, Baltimore, MD, United States; ^5^ Institute of Marine and Environmental Technology, Baltimore, MD, United States; ^6^ Laboratory for Marine Biology and Biotechnology, Qingdao National Laboratory for Marine Science and Technology, Qingdao, China

**Keywords:** cartilaginous fishes, shark, T cells, CD8, MHC, structure, evolution

## Abstract

**Introduction:**

Cartilaginous fishes are the most evolutionary-distant vertebrates from mammals and possess an immunoglobulin (Ig)- and T cell-mediated adaptive immunity. CD8 is the hallmark receptor of cytotoxic T cells and is required for the formation of T cell receptor-major histocompatibility complex (TCR-MHC) class I complexes.

**Methods:**

RACE PCR was used to obtain gene sequences. Direct dilution was applied for the refolding of denatured recombinant CD8 protein. Hanging-drop vapor diffusion method was performed for protein crystallization.

**Results:**

In this study, CD8α and CD8β orthologues (termed ScCD8α and ScCD8β) were identified in small-spotted catshark (*Scyliorhinus canicula*). Both ScCD8α and ScCD8β possess an extracellular immunoglobulin superfamily (IgSF) V domain as in previously identified CD8 proteins. The genes encoding CD8α and CD8β are tandemly linked in the genomes of all jawed vertebrates studied, suggesting that they were duplicated from a common ancestral gene before the divergence of cartilaginous fishes and other vertebrates. We determined the crystal structure of the ScCD8α ectodomain homodimer at a resolution of 1.35 Å and show that it exhibits the typical topological structure of CD8α from endotherms. As in mammals, the homodimer formation of ScCD8αα relies upon interactions within a hydrophobic core although this differs in position and amino acid composition. Importantly, ScCD8αα shares the canonical cavity required for interaction with peptide-loaded MHC I in mammals. Furthermore, it was found that ScCD8α can co-immunoprecipitate with ScCD8β, indicating that it can form both homodimeric and heterodimeric complexes.

**Conclusion:**

Our results expand the current knowledge of vertebrate CD8 dimerization and the interaction between CD8α with p/MHC I from an evolutionary perspective.

## Introduction

The divergence of jawless and jawed (gnathostome) vertebrates is a major event in evolution, which was accompanied by drastic morphological and physiological changes (1, 2). Of note, all gnathostomes are equipped with an antibody-based adaptive immune system orchestrated by immunoglobulins (Igs) and T cell receptors (TCRs), which are somatically rearranged by the recombination-activating genes (RAGs), and polymorphic/polygenic major histocompatibility complex (MHC) molecules, which are used to fend off pathogen evasion (3–5).

CD8^+^ T cytotoxic cells are key immune cells in the adaptive immune response and are vital for limiting the replication of intracellular pathogens such as viruses by killing the infected cells. These cells are activated by peptides presented by MHC class I molecules (p/MHC I) and secret cytotoxins such as perforin and granzymes to induce apoptosis in the target cells. CD8αα-p/MHC I complexes interact with TCR/CD3 complexes to transduce signals within the T cells. To ensure efficient T cell activation, CD8 acts as a co-receptor to stabilize the interaction of TCR with MHC I on the surface of antigen-presenting cells (APCs) (6, 7). The CD8 molecule is a type I membrane glycoprotein with two isoforms, CD8α and CD8β, which can form homodimeric (CD8αα) or heterodimeric (CD8αβ) complexes (8). Both CD8α and CD8β have an extracellular Ig superfamily (IgSF) V domain, a stalk region, a transmembrane domain and an intracellular cytoplasmic tail which contains a CxC/Cxx motif for binding to the Src kinase p56^Lck^ to elicit cellular responses (9, 10). CD8α and CD8β are structurally related and the genes encoding them are tandemly linked in the genomes in both mammals and bony fishes (11). Although CD8αα and CD8αβ bind with similar affinities to p/MHC I and both can be recruited to the immunological synapse, their expression differs between immune cell types. CD8αα is found on γδ T cells, NK cells, subsets of dendritic cells and intestinal intraepithelial lymphocytes, whilst CD8αβ expression is limited to αβ T cells (6, 12). To date, the functions of CD8αα are still not fully characterized. However, it has been shown that CD8αα can bind non-classical MHC I molecules with greater affinity than classical MHC I molecules (13, 14), helping determine the differentiation of memory and mucosal T cell immune responses. Additionally, recent studies suggest that CD8αα may function as a negative regulator for T cell activation (15). The crystal structures of CD8αα homodimer and CD8αα/p/MHC I complex have been solved in mammals and birds (16–18). The CD8αα homodimer consists of two symmetric IgSF V domains, with several highly conserved residues being critical for CD8α dimerization and interaction between CD8α and p/MHC I molecule. Recently, we solved the crystal structure of grass carp CD8αα (19) and found that it has several unique topological features. For instance, the cavity of human CD8αα which is shaped by the CDR loops and forms the principal area of interaction with p/MHC I, is absent in grass carp CD8αα. However, this appears to have little impact upon the binding with peptide-Ctid-UAA-β2m (p/UAA-β2m) binding (20), suggesting that the mechanisms of interactions between CD8αα and p/MHC I in exothermic vertebrates may differ from that in endotherms.

The cartilaginous fishes (Chondrichthyes: sharks, skates, rays, and chimaeras) diverged from other jawed vertebrates approximately 450 million years ago and are the most ancient vertebrate lineage to possess a canonical adaptive immune system. It has been shown that the protein structure of elephant shark (*Callorhinchus milii*) MHC I molecule is structurally conserved (21). Analyses of the elephant shark draft genome assembly confirmed that the *CD8α* and *CD8β* genes are present in cartilaginous fishes (2, 22). In this study, we sequenced *CD8α* and *CD8β* from small-spotted catshark (*Scyliorhinus canicular*; *Sc*), and show that the encoded proteins possess an IgSF V domain and that CD8α could form homodimer and heterodimer with CD8β. Moreover, the structure of the *Sc*CD8αα extracellular region was solved, revealing that CD8α IgSF V domain contains a canonical cavity which is present in the interface with p/MHC I in mammals. Our study provides the first evidence that cartilaginous fish CD8α can form homodimer through a conserved hydrophobic core, and that CD8α can interact with CD8β.

## Materials and methods

### Animals

Captive-bred, small-spotted catsharks of approximately three years of age, were maintained in artificial seawater at 8-12°C in indoor tanks at the University of Aberdeen, UK. Animals were overdosed in MS-222 prior to sacrifice and tissue harvest. All procedures were conducted in accordance with UK Home Office ‘Animals and Scientific Procedures Act 1986; Amendment Regulations 2012’ on animal care and use, with prior ethical approval from the University of Aberdeen’s Animal Welfare and Ethical Review Body (AWERB). Tissue samples were homogenized in TRIzol reagent (Sigma-Aldrich) using a TissueLyser II (Qiagen) and total RNA isolation performed according to standard protocols. RNA samples were quantified using the Agilent 2100 Bioanalyzer (Agilent) and were pooled for subsequent gene cloning (23).

### Identification and cloning of *Sc*CD8

Partial cDNA sequences of *Sc*CD8α and *Sc*CD8β were obtained from the NCBI *Scyliorhinus* database (https://www.ncbi.nlm.nih.gov/) using the local BLAST search tool and the top hits used to design gene-specific primers (GSP) for cloning. The full-length cDNA sequences were obtained using GSP by rapid amplification of cDNA ends (RACE) PCR. For 5′ RACE, first strand cDNA was tailed with dCTPs and used as template. The first round PCR was performed using a Premix Ex Taq™ Hot Start Mix (TaKaRa) and primer pairs, 5R1/APG. Primers 5R2/AP were used for the second round PCR of 5′ RACE. For the 3’ RACE, primers 3F1/APT and 3F2/AP were used for the first and second round PCR, respectively (24). 5′ and 3′ RACE was performed as described previously (25). Briefly, the first round PCR was performed under the following conditions: 1 cycle of 95°C/3 min; 35 cycles of 98°C/10 s, 60°C/30 s, 72°C/1 min; 1 cycle of 72°C/7 min. The first round PCR products were diluted (1:50, v/v) and used as template for the second round PCR and cycling conditions are: 1 cycle of 95°C/3 min; 5 cycles of 98°C/10 s, 70°C/30 s, 72°C/1 min; 5 cycles of 98°C/10 s, 68°C/30 s, 72°C/1 min; 5 cycles of 98°C/10 s, 66°C/30 s, 72°C/1 min; 5 cycles of 98°C/10 s, 64°C/30 s, 72°C/1 min; 5 cycles of 98°C/10 s, 62°C/30 s, 72°C/1 min; 10 cycles of 98°C/10 s, 60°C/30 s, 72°C/1 min;1 cycle of 72°C/7 min.

PCR products were sequenced and assembled into the full-length cDNA sequences using the DNAMAN program (version 6.0). The complete coding sequences (CDS) were verified by sequencing the PCR products amplified using one pair of primers located at the 5’ and 3’ untranslated region (UTR). Primers are described in **Table 1**.

**Table 1:** Primers used in this study.

Primers	Sequence (5′ to 3′)	Application
CD8α-F1	CAAAGTACACGGGTGGAGGAAGG	3′-RACE
CD8α-F2	TATATCTGGCATCGGTTCACAAAAT	3′-RACE
CD8α-R1	GGTGACTTTTTGGGATGATTTGTCA	5′-RACE
CD8α-R2	TCAGCCATGAGCGAGCAGGAGA	5′-RACE
CD8β-R1	TTGCGACTGATGTTCGGGTA	5′-RACE
CD8β-R2	GCGACTGATGTTCGGGTACT	5′-RACE
CD8α-F	TCTCAAGTGCCCTCGTTCAC	Verify full length
CD8α-R	ATTGAGCTCTCAAGGGGTGC	Verify full length
CD8β-F	GGTCTTCAGCAAACAGCAACA	Verify full length
CD8β-R	TCCTTTCTCCACATGTTGGTCC	Verify full length
r*Sc*CD8α-F	CATGCCATGGCTAGCCTGCAGAGCACCCGTGTTGAAGAAGGTTCCAAAGTTGCCAT	Plasmid construction
r*Sc*CD8α-R	CGGGATCCTTACATGGCACAGGACAGCCCATCTTG	Plasmid construction
APT	CCAGACTCGTGGCTGATGCATTTTTTTTTTTTTTTT	3′-RACE
APG	CCAGACTCGTGGCTGATGCAGGGGGGGGGGGGGG	5′-RACE
AP	CCAGACTCGTGGCTGATGCA	5′- and 3′-RACE

### Sequence analysis of *Sc*CD8α and *Sc*CD8β

The cDNA and protein sequences of *Sc*CD8α and *Sc*CD8β were predicted using the Genetyx program. Signal peptides, molecular weight and isoelectric point (pI) were predicted using software available on the ExPasy server (https://www.expasy.org/). Known CD8α sequences were retrieved from the NCBI GenBank database. Amino acid (aa) identity between the sequences was determined using the EMBOSS Tools (https://www.ebi.ac.uk/Tools/psa/emboss_needle/). The phylogenetic tree was constructed using the Neighbor-Joining method of MEGA X program and repeated for 10,000 times to obtain the bootstrap scores. The signal peptide cleavage site was predicted using the SignalP 5.0 program (http://www.cbs.dtu.dk/services/SignalP/).

### Production and purification of recombinant *Sc*CD8α protein

The cDNA fragment encoding the extracellular IgSF V domain of *Sc*CD8α (Ser20-Met163) was amplified and cloned into the expression vector pET21d between the *Nco* I and *BamH* I sites, with the codons for the first ten aa optimized for expression in the *E. coli*. The recombinant pET21d-*Sc*CD8α plasmid was transformed into *E. coli* Rosetta (DE3) cells. The recombinant *Sc*CD8α protein was expressed as inclusion bodies after induction with 0.5 mM IPTG. The inclusion bodies were collected as previously described (19), and denatured in 6 M guanidine hydrochloride buffer (6 M guanidine hydrochloride, 50 mM Tris-HCl (pH 8.0), 10 mM EDTA, 100 mM NaCl, 10% glycerol (v/v) and 10 mM DTT) (19). The protein concentration was adjusted to 30 mg/ml prior to protein refolding.

Dilution renaturation was used to obtain soluble *Sc*CD8α (19). Briefly, 5 ml of *Sc*CD8α-containing denaturing solution (at 30 mg/ml) was slowly dropped into 500 ml of refolding buffer (100 mM Tris-HCl, 2 mM EDTA, 400 mM L-arginine-HCl, 0.5 mM oxidized glutathione and 5 mM reduced glutathione, pH 8.0) at 4°C and slowly stirred for 24 h. The resulting protein solution was then concentrated using a 10 kDa cutoff filter and buffer-exchanged into 10 mM Tris-HCl with 50 mM NaCl (pH 8.0). Concentrated protein was then subject to size exclusion chromatography over a Superdex 200 16/600 column (GE Healthcare) and polished by passage over a Resource Q anion-exchange chromatography column (GE Healthcare). Protein fractions were collected at 1 ml per tube and checked by SDS-PAGE. Purified *Sc*CD8α protein was again buffer-exchanged into 20 mM Tris-HCl with 50 mM NaCl (pH 8.0). The recombinant *Sc*CD8α was also checked by PAGE under non-reducing conditions. PAGE was run in Tris-Gly buffer (pH 4.4, WSHTBio, China) in an ice-water bath at 120 V for 2 h.

### Co-immunoprecipitation

The coding sequences of *Sc*CD8α and *Sc*CD8β were synthesized with a C-terminal Myc-tag or HA-tag and cloned into pcDNA3.1 (GENEWIZ, China). HEK293 cells were grown in 25cm^2^ flasks to reach 80-90% confluence and transfected with 5 μg of each plasmid using the jetOPTIMUS^®^ reagent (polyplus). After 24 h, the transfected HEK293 cells were collected and lysed in the radioimmunoprecipitation assay (RIPA) buffer (Beyotime, China) containing 1% phenylmethanesulfonyl fluoride (PMSF, Beyotime) on ice for 30 min. The lysed sample was centrifuged at 12,000 g for 15 min to pellet cellular debris and the supernatant collected into a clean 1.5 ml microtube. Following the removal of 50 μl for Western blot analysis, 40 μl of α-Myc-conjugated magnetic beads (Abmart) was added to the remaining supernatant and incubated overnight on a shaker. The magnetic beads were collected by centrifugation at 1,500 g for 3 min at 4°C, washed 3 times with ice-cold PBS, resuspended in 30 µl 2 × SDS loading buffer and prepared for Western blot analysis as detailed description previous (26). Briefly, Protein samples are separated by SDS-PAGE, then transferred to PVDF membranes using a semi-dry method. The membrane is blocked with a TBS buffer containing 5% non-fat milk for 1 h at room temperature. It is then incubated overnight at 4°C with the α-Myc or α-HA (1:1000, v/v, Huabio, China). After washing with TBS-T buffer 3 times, the membrane was incubated with a goat α-mouse IgG H&L antibody (IRDye^®^ 680RD, 1: 10,000 dilution, v/v, Odyssey, USA), for 1 h in the dark. The membrane was washed as above then imaged using the Odyssey CLx image system (Odyssey, USA).

### Protein crystallization

Purified *Sc*CD8α protein was concentrated to 5 mg/ml or 10 mg/ml and an initial crystallization screen performed by the hanging-drop vapor diffusion method at 277 K. The Wizard™ Classic line of random sparse matrix screening (Rigaku Reagents) was used to establish crystallization conditions where the protein solution was mixed with reservoir buffer at a 1:1 ratio (v/v). The *Sc*CD8α crystals formed in Wizard classic 1, Formulation 41 (30% (w/v) PEG3000 and 100 mM CHES/Sodium hydroxide pH 9.5) after 5 days at a protein concentration of 10 mg/ml.

### Collection and processing of diffraction data

Diffraction data on the *Sc*CD8α crystals were collected on beamline BL19U1 at the wavelength of 0.97923 Å, with an ADSC 315 CCD detector at the Shanghai Synchrotron Radiation Facility (SSRF), China. The crystals were soaked for several seconds in a reservoir solution containing 15% glycerol as a cryoprotectant and then flash-cooled in a stream of gaseous nitrogen at 100 K. The collected intensities were indexed, integrated, corrected for absorption, scaled and merged using HKL 3000.

### Structural analysis

The structure of *Sc*CD8α was determined by molecular replacement with the MOLREP and PHASER programmer using the chicken CD8α protein structure (Protein Data Bank [PDB] code: 5EB9) as a search model. A comprehensive model was built manually with COOT (27), and the structure was refined with the REFMAC 5 program. Refinement rounds were implemented using PHENIX as previously described (28), and the stereochemical quality of the final model was assessed by the PROCHECK program. Details of data collection and refinement are shown in **Table 2**. PyMOL was used to generate and visualize the structural figures. Multiple sequence alignment was performed by the Clustal Omega server (https://www.ebi.ac.uk/Tools/msa/clustalo/) and ESPript 3.0 (http://espript.ibcp.fr/ESPript/ESPript/). Scores for accessible surface area (ASA) and buried surface area (BSA) were calculated using the PDBePISA server (http://www.ebi.ac.uk/msd-srv/prot_int/pistart.html).

**Table 2:** Data collection and refinement statistics (molecular replacement).

	Crystal Data
Data collection	ScCD8α
Space group	C121
Cell dimensions	
*a*, *b*, *c* (Å)	72.38, 40.45, 32.83
α,; β,; γ (°)	90, 92.56, 90
Resolution (Å)	35.3-1.35
*R*_merge_	0.03
*I/*σ*I*	2.19 (at 1.35 Å)
Completeness (%) (in resolution range)	99.2 (35.3-1.35)
Redundancy	12.4 (13.0)
Wavelength	0.97923 Å
Beamline	BL19U1
Refinement	
Resolution (Å)	35.3-1.35
No. reflections	1113
*R*_work_/*R*_free_	0.198/0.2
No. atoms	919
Protein	100
Ligand/ion	
Water	125
*B*-factors	17.0
R.m.s. deviations	
Bond lengths (Å)	0.49
Bond angles (°)	0.66
Ramachandran statistics	
Most favored (%)	98
Disallowed (%)	0.0

*Values in parentheses are for highest-resolution shell.

## Results

### The *CD8α* and *CD8β* genes had diverged in cartilaginous fishes

In the present study, CD8α and CD8β were identified from small-spotted catshark. The full-length cDNA sequence of the *ScCD8α* (NCBI accession number: MT840192) is 917 bp with an open reading frame (ORF) of 657 bp and encodes a protein of 218 aa with a putative signal peptide of 19 aa. A CQH motif is present in the cytoplasmic tail of *Sc*CD8α permitting its interaction with Lyk kinase. Three cysteine residues are present in the ectodomain, two of which are conserved in all previously characterized CD8α molecules (**Supplementary Figure 1**). The obtained cDNA sequence of *ScCD8β* (NCBI accession number: MW713126.1) is 726 bp containing an ORF of 648 bp and encodes a protein of 215 aa with a signal peptide of 19 aa. It has a 5′-UTR of 39 bp and a 3′-UTR of 42 bp (**Supplementary Figure 2**). Although *Sc*CD8α and *Sc*CD8β have low (20%-30%) aa identity with their orthologues from other species (**Supplementary Table 1**), they share a similar domain structure (i.e., IgSF V-transmembrane region-cytoplasmic tail).

**Figure 1:**
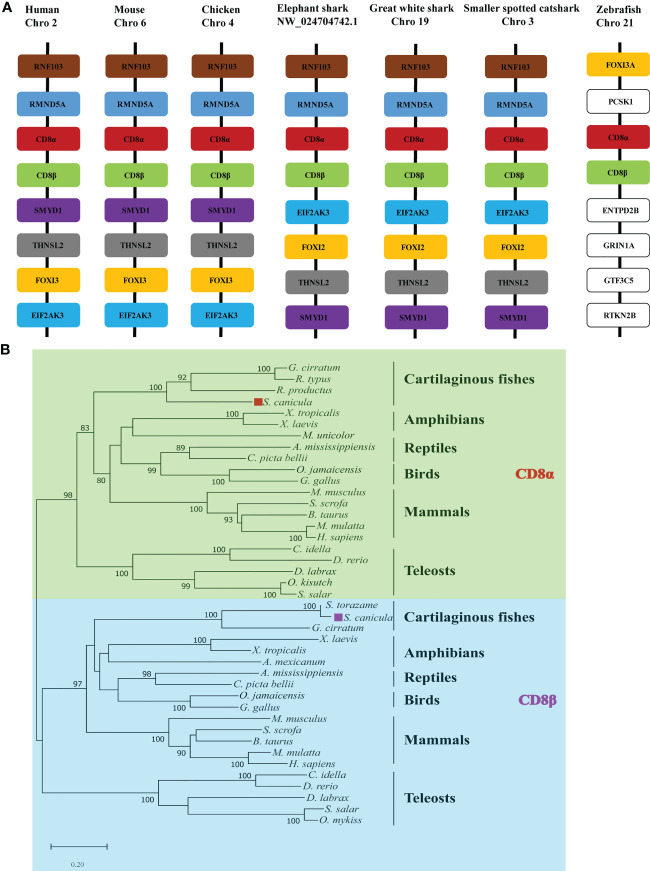
Analysis of *ScCD8* genes. **(A)** Gene synteny analysis of CD8α, the synteny information for human, mouse, chicken, elephant shark, great white shark, zebrafish and small-spotted catshark, and spotted gar genes were obtained from the Ensembl database. **(B)** Neighbor-joining (N-J) phylogenetic tree of *CD8* genes. The tree was constructed using the N-J method within the MEGA-X program with bootstrap values shown next to the branches based on 10,000 bootstrap replications. “◼” indicates *Sc*CD8α (red) and *Sc*CD8β (purple). The accession numbers of protein sequences are provided in **Supplementary Table 1**.

Gene synteny for cartilaginous fish CD8α and CD8β genes was determined using the draft genomes for elephant shark, white shark (*Carcharodon carcharias*), and small-spotted catshark (**Figure 1A**). This showed that the *CD8α* and *CD8β* genes are tandemly linked in all three species and are sandwiched by the *RMND5A* and *FOX12/13* genes. This synteny is conserved in chicken, mouse, and human (**Figure 1A**). The CD8 genes are also arranged in tandem in zebrafish, while the surrounding genes are not conserved. To determine the phylogenetic relationships of *Sc*CD8α and *Sc*CD8β, an unrooted Neighbor-Joining tree was constructed using CD8α and CD8β protein sequences from cartilaginous fishes, teleosts, amphibians, reptiles, birds and mammals. As shown in **Figure 1B**, the CD8α and CD8β clades are well separated, suggesting that the duplication event generating two CD8 genes occurred before the divergence of cartilaginous fishes and other vertebrates. In line with many other studies, cartilaginous fish CD8α and CD8β grouped with their tetrapod orthologs (node bootstrap values of 83% and 97% respectively), while teleost CD8αs and CD8βs form an independent clade. This is likely a consequence of the extra round(s) of genome wide duplication experienced by teleost fishes (29) and the higher evolutionary rate of both protein-coding and non-coding sequences in this lineage compared to other vertebrates (30).

### The topological structure of *Sc*CD8αα

**Figure 2:**
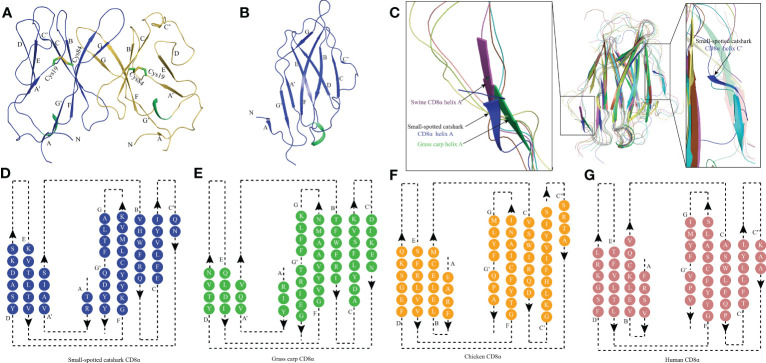
The topological structure of *Sc*CD8αα extracellular region. **(A)** Cartoonstructure of the *Sc*CD8αα homodimer, CD8α monomers are colored blue and orange. The β-strands are labelled. Disulfide bonds are shown as sticks and colored lime green. **(B)** The monomer structure of the *Sc*CD8α extracellular region. **(C)** Comparison of *Sc*CD8α extracellular region to that of other species. All the structures are shown in cartoon representation. The *Sc*CD8α is colored blue, grass carp CD8α is lime green, chicken CD8α is yellow, bovine CD8α is cyan, human CD8α is pink, mouse CD8α is orange, monkey CD8α is limon, and swine CD8α is light magenta. Helix A of *Sc*CD8α, swine CD8α, and grass carp CD8α are labeled. Helix C’ of *Sc*CD8α is labeled in the righthand panel. PDB accession numbers for each structure are as follows; human CD8αα 1CD8, chicken CD8αα 5EB9, swine CD8αα 5EDX, grass carp CD8αα 5Z11, monkey CD8αα 2Q3A, mouse CD8αα 2ARJ and bovine CD8αα 5EBG. **(D-G)** Two-dimensional topology diagrams of vertebrate CD8αs. The strands form a sandwich of 2 sheets in the catshark, grass carp, chicken, and human, which are colored blue, lime green, orange, and pink respectively, β-strands are labeled.

The recombinant protein of the *Sc*CD8α extracellular region expressed in bacteria and purified (**Supplementary Figure 3**). SDS-PAGE analysis under reducing conditions revealed a single band of approx. 16 kDa. However, PAGE analysis under non-reducing conditions showed a protein band of approx. 32 kDa, indicating that it exists as a dimer (**Supplementary Figure 3C**). The structure of recombinant *Sc*CD8α extracellular region (**Supplementary Figure 2**) was solved at a resolution of 1.35 Å with a space group of C121 and further refined to a R_work_/R_free_ factor of 0.198/0.2 (**Table 2**). The structure is composed of two molecules arranged in the asymmetric unit. Despite low sequence homology with known CD8α molecules, *Sc*CD8αα retains typical IgSF V architecture. Like its counterparts in higher vertebrates, the *Sc*CD8α dimer consists of 2 anti-parallel β sheets containing 10 β strands (**Figure 2A**), labeled as A, A’, B, C, C’, D, E, F, G, G’. The front sheet is occupied by strands A’, D and E, while the back sheet contains strand A, B, C, C’ F, G and G’ (**Figures 2A, B**). Notably, the A strand is in the back β sheet, lying parallel to strand G’ (**Figures 2C, D**), an arrangement seen only in grass carp CD8α thus far. In contrast, the A strand of chicken and human CD8αs is in the front sheet and lies in parallel to the B strand (**Figures 2E–G**). Two cysteines (Cys19 and Cys84) form a disulphide bond linking the front and back sheet of *Sc*CD8α and stabilizing the IgSF V architecture (**Figure 2A**). The RMSD values of *Sc*CD8αα with known CD8αα from other species were determined, including human (1.63), monkey (1.70), mouse (1.70), bovine (2.56), swine (2.10) chicken (1.54), and grass carp (1.66) (**Supplementary Figure 4**).

**Figure 3:**
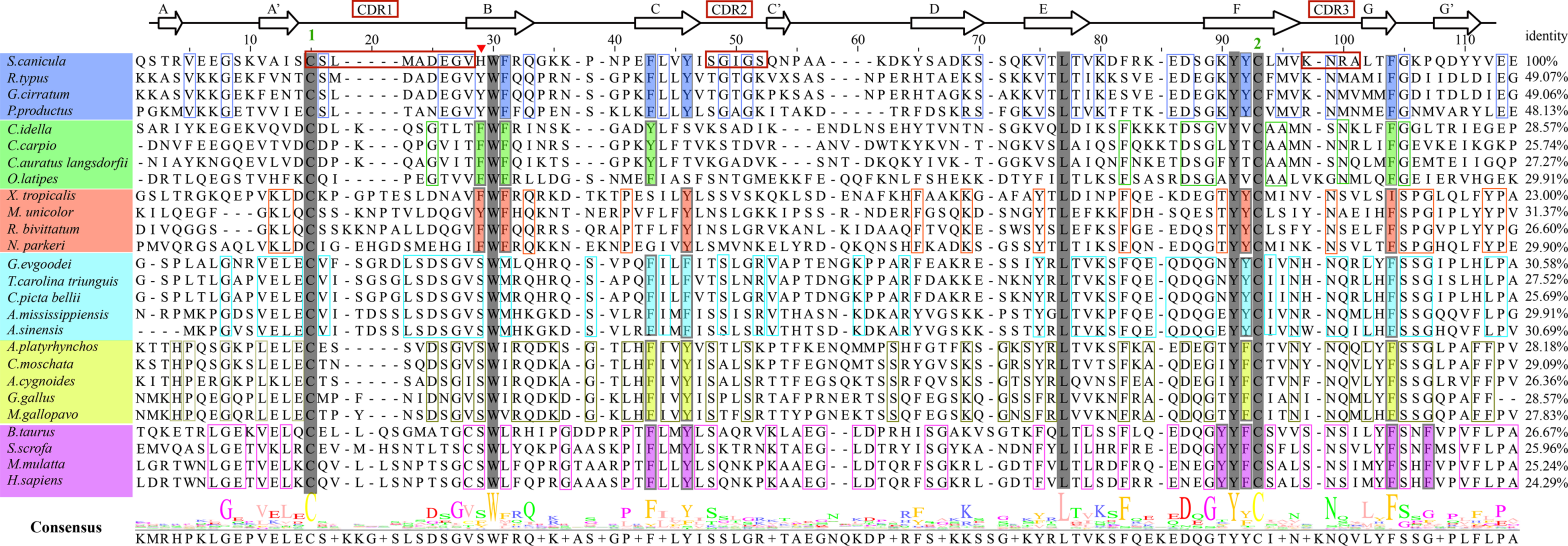
Amino acid sequence alignment of *Sc*CD8α with CD8αs from different vertebrate species, with secondary structure elements indicated. Black arrows above the alignment indicate β-strands. Green numbers denote cysteines that form disulfide bonds, CDRs are outlined with red boxes. The residues conserved in the sampled species are shaded gray. The aa residues conserved in specific vertebrate lineages are marked with colored boxes. The hydrophobic core or predicted core are highlighted with colored rectangles. Amino acid identities between *Sc*CD8α and the listed CD8α molecules are shown at the end of each sequence. The consensus sequence is shown at the bottom of the alignment. His28 in ScCD8α IgSF V domain is indicated by “▾”.

To understand how CD8α has maintained core structural features during evolution, structure-based sequence alignment of selected vertebrate CD8α molecules was performed (**Figure 3**). Unsurprisingly, *Sc*CD8α shares relatively higher aa identities with CD8α homologs from cartilaginous fish CD8α (48.1-49.1%) than with those from bony fish and tetrapods, for instance, 24.3% with human CD8α, 28.6% with chicken CD8α and 28.6% with grass carp CD8α. The disulfide bond in the IgSF V domain, known to be vital for maintaining the overall structure of CD8α, is conserved in all the species examined (**Figures 2**, **3**). Also conserved are Trp30*^Sc^* ^CD8α^, Leu77*^Sc^* ^CD8α^, Tyr91*^Sc^* ^CD8α^ and Phe104*^Sc^* ^CD8α^, which constitute the backbone of CD8α for stabilizing conformation.

### *Sc*CD8α maintains dimerization through a distinctive hydrophobic core

**Figure 4:**
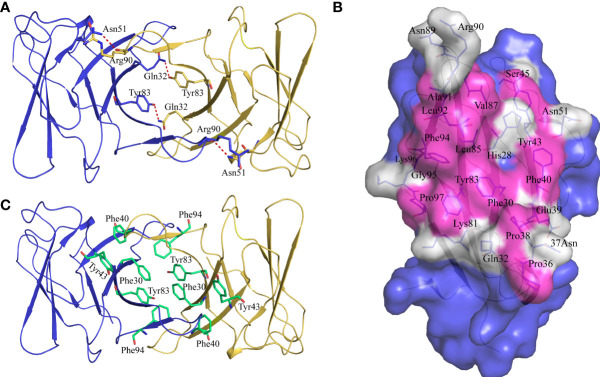
Characterization of the intermolecular interactions that maintain the dimerization of ScCD8αα. **(A)** The inter-chain hydrogen bonds of *Sc*CD8αα. The *Sc*CD8αα is shown in cartoon representation with the monomers colored blue and orange. Side chains that assist dimerization through the formation of hydrogen bonds are shown as stick models with their amino acid and position labeled. Hydrogen bonds are shown as red dashed lines. **(B)** The residues in the interface of the *Sc*CD8αα. *Sc*CD8α is surface rendered in blue. The residues forming the interface are shown as sticks and surface rendered in white while hydrophobic residues are surface rendered in magenta. Amino acid residues and their positions are labeled. **(C)** Side chains forming the hydrophobic core of *Sc*CD8αα. The backbone of *Sc*CD8αα is shown as a cartoon with the monomers colored blue and orange. The residues forming the hydrophobic core are shown as stick models and colored lime green. Amino acid residues and their positions are labeled.

**Figure 5:**
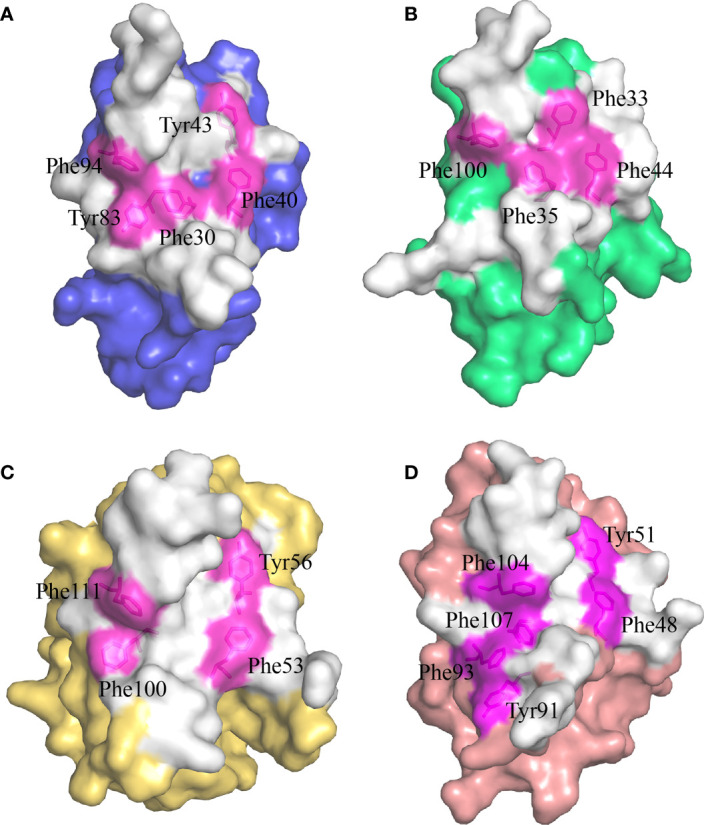
The hydrophobic core of the CD8αα homodimer in small-spotted catshark compared to other vertebrate species. The surface of each CD8α monomer is rendered in a different color while residues forming the hydrophobic core are shown in stick representation and surface rendered in magenta. **(A)** The hydrophobic core of *Sc*CD8αα is formed by five hydrophobic residues (Phe30, Tyr43, Phe40, Tyr83 and Phe94). **(B)** The hydrophobic core of grass carp CD8αα is formed by four hydrophobic residues (Phe32, Phe35, Tyr44, and Phe100). **(C)** The hydrophobic core of chicken CD8αα is formed by four hydrophobic residues (Phe53, Tyr56, Phe100, and Phe111). **(D)** The hydrophobic core of human CD8αα is formed by six hydrophobic residues (Phe48, Tyr51, Tyr91, Phe93, Phe104 and Phe107).

Previous studies have shown that stabilization of the CD8αα homodimer relies upon a hydrophobic core which forms at the interface of the two CD8α monomers and is composed of several hydrophobic aromatic residues (18, 19). The *Sc*CD8αα interface contains four inter-chain hydrogen bonds, which are formed by Gln32 (chain A) and Tyr83 (chain B), Asn51 (chain A) and Arg90 (chain B), Gln32 (chain B) and Tyr83 (chain A), Asn51 (chain B) and Arg90 (chain A), respectively (**Figure 4A**). Strands B, C, C’, F, G and G’ in the back sheet of *Sc*CD8α are heavily involved in dimerization, with a buried surface area (BSA) of 853.3 Å^2^. In the *Sc*CD8αα interface, 23 residues are involved in dimerization, 12 of which are hydrophobic, burying a surface area of 541.29 Å^2^ and making a major contribution (63.4%) to the van der Waals interactions (**Figure 4B** and **Table 2**). Five aromatic residues including Phe30 (strand B), Phe40 and Tyr43 (strand C), Tyr83 (strand F) and Phe94 (strand G) are located in the center of *Sc*CD8αα dimer, forming a hydrophobic core with a BSA of 315.51 Å^2^ and contributing to 58.2% of the total hydrophobic force (**Figures 4C**, **5A**). Interestingly, the composition and spatial distribution of these 5 aromatic residues are conserved in all cartilaginous fish CD8αs but not in CD8αs from bony fishes, birds, and mammals (**Figures 5B–D**).

### ScCD8α can form a heterodimer with ScCD8β

**Figure 6:**
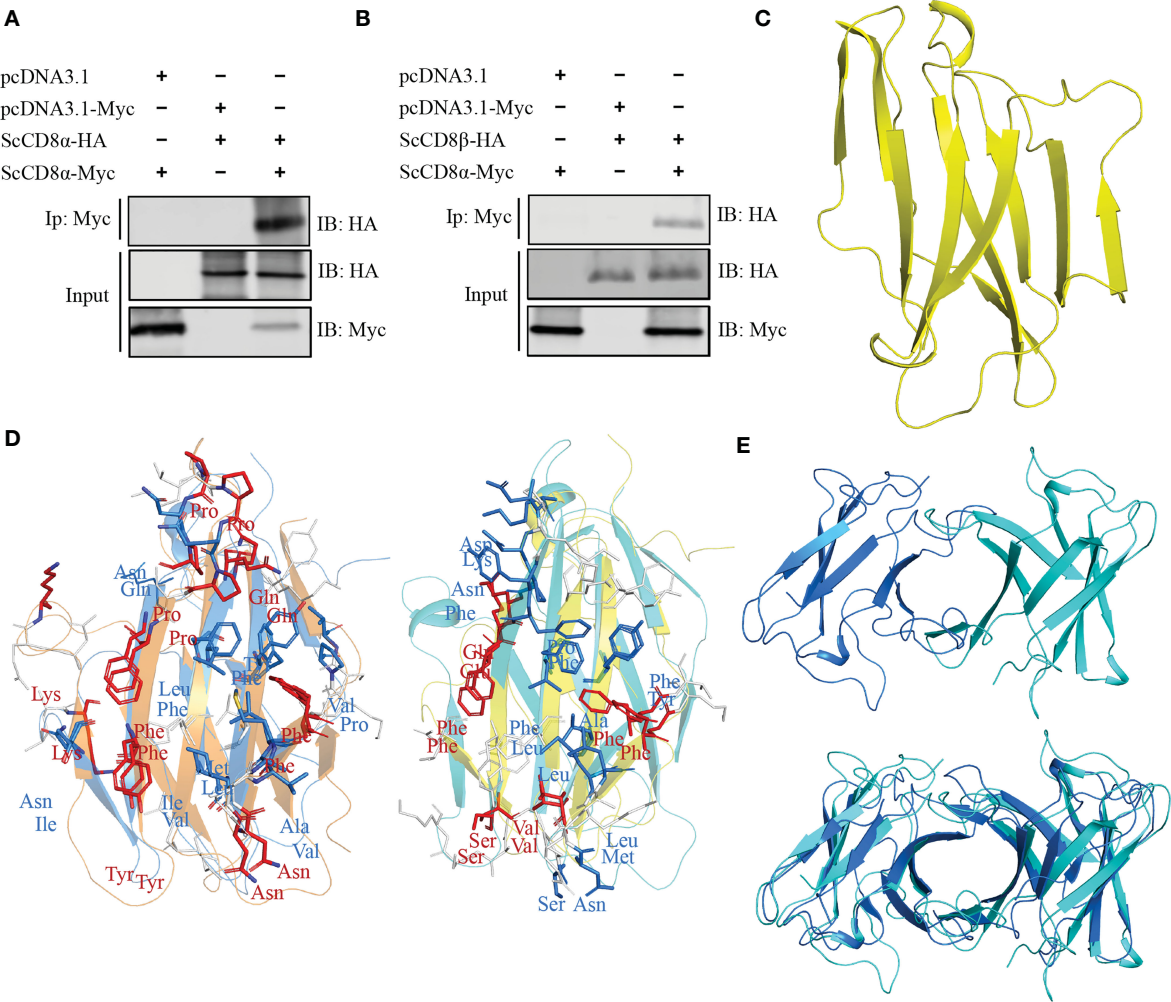
Interaction and modelling and of *Sc*CD8α and *Sc*CD8β. **(A, B)** Interaction of *Sc*CD8α with *Sc*CD8α **(A)** and *Sc*CD8β **(B)**. HEK293 cells were co-transfected with the indicated expression plasmids (5 μg each). After 24h, cell lysates were immunoprecipitated (IP) with α-Myc Ab conjugated agarose beads, and immunoblotted (IB) was detected with α-Myc or α-HA Abs. **(C)** Model of *Sc*CD8β shown as a cartoon structure. **(D)** Predicted interface of CD8αβ with the *Sc*CD8α monomer shown in marine, *Sc*CD8β in cyan, mouse CD8α in orange, and CD8β in yellow. The side chains of key residues forming the CD8αβ interface are shown as sticks. Conserved residues are colored red, physiochemically-conserved residues are colored marine, other residues are shown in white. **(E)** The structural model of *Sc*CD8αβ (top) and shown structurally aligned with *Sc*CD8αα (bottom). *Sc*CD8α is colored marine and *Sc*CD8β cyan.

To explore whether *Sc*CD8α can also interact with *Sc*CD8β, we co-transfected HEK293 cells with *Sc*CD8α-Myc and *Sc*CD8α-HA or *Sc*CD8α-Myc and *Sc*CD8β-HA then immunoprecipitated (IP) any complexes formed with an agarose bead conjugated α-Myc antibody. Our results show that both HA-tagged *Sc*CD8α (**Figure 6A**) and HA-tagged *Sc*CD8β (**Figure 6B**) can be immunoprecipitated with Myc-tagged *Sc*CD8α using an α-Myc antibody. This indicates that both CD8αα homodimer and CD8αβ heterodimer can be formed in small-spotted catshark. To investigate this further, we modelled the 3D structure of *Sc*CD8β extracellular region (Val22-Val132, without signal peptide) using human immunoglobulin lambda light chain (PDB code: 6ID4) as a search model, and showed that it also exhibits typical IgSF V architecture (**Figure 6C**).

The structure of the mouse CD8αβ (mCD8αβ) heterodimer structure (PDB code: 2ATP) indicates that dimerization of CD8αβ is maintained by hydrophobic interactions as seen in CD8αα (10). We superposed the structures of *Sc*CD8α and *Sc*CD8β with the mouse CD8αβ structure (**Figure 6D**). We observed that 8 residues of CD8α within the interface are identical between the two structures and 8 residues are physico-chemically similar. Similarly, five identical residues and 8 physico-chemically conserved residues in the interface of CD8αβ structure can be identified in *Sc*CD8β and mCD8β (**Figure 6D**). Furthermore, structural superposition revealed striking resemblance between *Sc*CD8αα and *Sc*CD8αβ (**Figure 6E**).

### *Sc*CD8αα is capable of interacting with *Sc*MHC I

**Figure 7:**
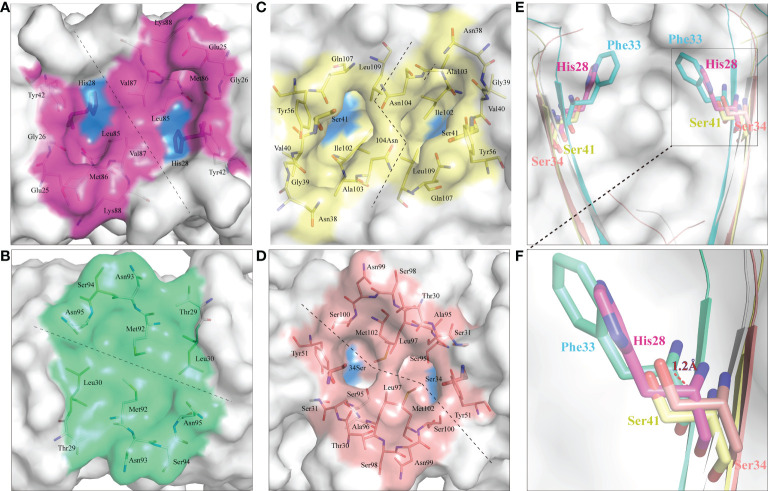
*Sc*CD8αα possess a canonical binding cavity. The composition of the binding cavities in representative vertebrate CD8αα structures are shown. Molecular surfaces are rendered in white, with residues key to dimeric interaction highlighted blue. **(A)** The cavity of *Sc*CD8αα, the side chains of cavity residues are shown as sticks and colored magenta. **(B)** The cavity of grass carp CD8αα, the side chains of cavity residues are shown as sticks and colored lime green. **(C)** The cavity of chicken CD8αα, the side chains of cavity residues are shown as sticks and colored yellow. **(D)** The cavity of human CD8αα, the side chains of cavity residues are shown as sticks and colored pink. **(E, F)** Comparison of key amino acids in the cavity of CD8αα structure in selected species, the residues and the distance between His28 and Phe33 are labeled.

CD8α is a co-receptor of the TCR-MHC class I complex. Studies of human, mouse and chicken CD8α have shown that the cavity formed by the CDR loops of CD8α is the main area for interaction with p/MHC I. Similarly, *Sc*CD8αα has a typical cavity, composed of 8 residues (Glu25, Gly26, His28, Tyr42, Leu85, Met86, Val87 and Lys88) symmetrically arranged in the CDR loops of the two monomers (**Figure 7A**), resulting in an accessible surface area (ASA) of 319.96Å^2^. This is a much smaller ASA than CD8ααs of other vertebrate species studied so far (e.g., chicken 641.36 Å^2^, swine 676.96 Å^2^, cow 585.2 Å^2^, mouse 727.85 Å^2^, monkey 526.42 Å^2^ and human 527.73 Å^2^) (**Figure 7**). Previous studies have shown that Ser34^hCD8α^ is vital for maintaining the cavity while substitution of Ser34^hCD8α^ by Phe33 results in disappearance of the cavity in grass carp CD8αα (12, 18). In cartilaginous fishes, the corresponding residue is occupied by histidine or tyrosine which, like phenylalanine, have large side chains. Structure alignment showed that His28^ScCD8α^ is placed approximately 1.2Å away from the surface, which may explain the formation of the cavity (**Figures 7E, F**).

**Figure 8:**
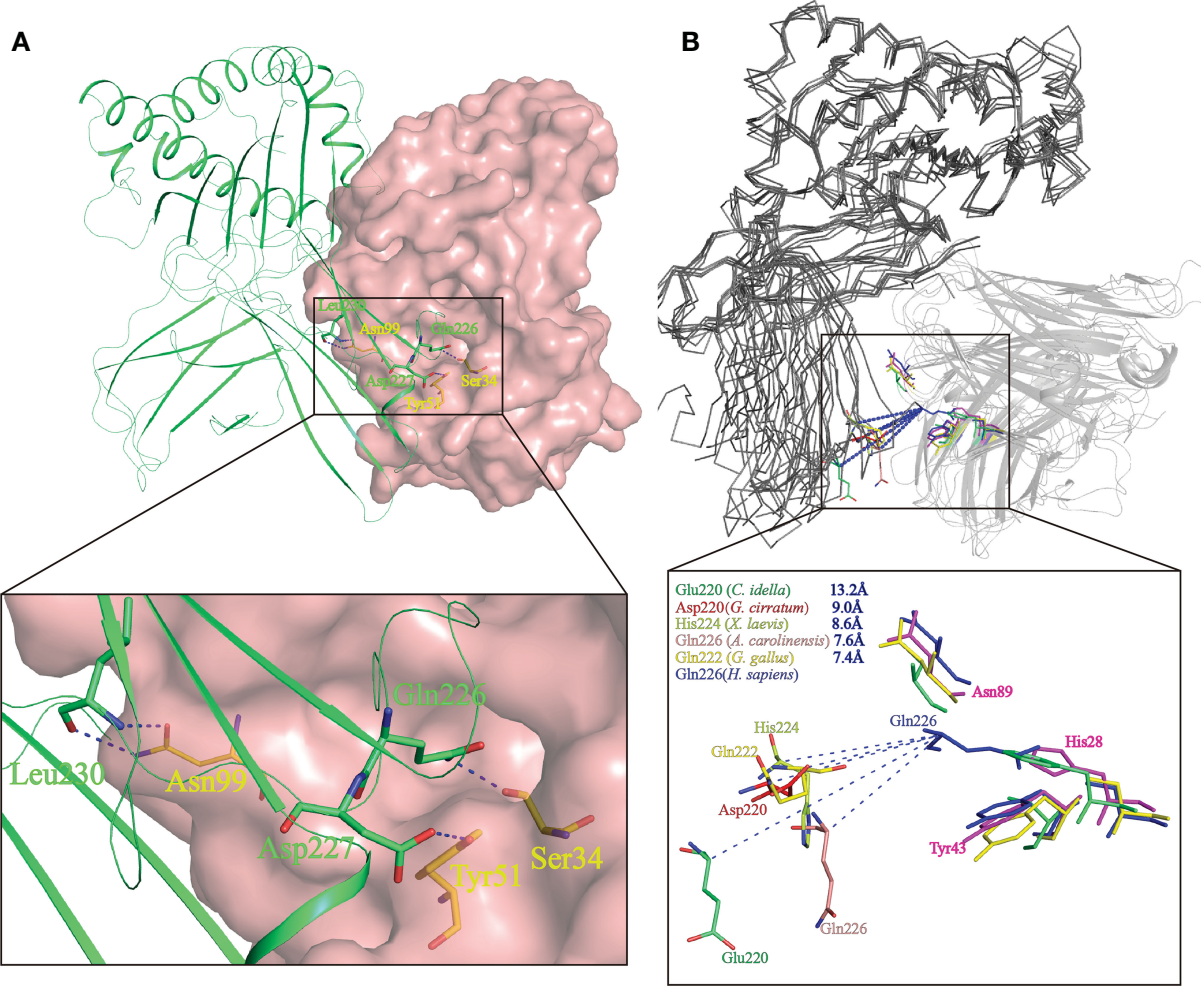
Prediction of the key intermolecular forces maintaining the interaction between *Sc*CD8αα and p/MHC I molecules. **(A)** The interaction between human CD8αα and p/MHC I Human MHC is shown as a lime green cartoon structure while the surface of human CD8αα is rendered in pink. The key amino acid side chains of the interaction between CD8αα (yellow) and p/MHC I (green) are shown. Key hydrogen bonds at the interface of human CD8αα and p/MHC I (PDB ID: 1AKJ) are indicated by blue dashed lines. **(B)** The shift distance between the CD loops reveals a different binding mode of CD8 in non-mammals. The MHC I molecules are shown as gray ribbons and the CD8αα structure as cartoon with a transparency of 50%. The shift distance is indicated by blue dash lines. Key residues in the CD loop of MHC I and the corresponding residues interacting with the CD8αα molecules are shown as sticks. Residues of MHC I and CD8αα of grass carp (limegreen), nurse shark (red), African clawed frog (limon), chicken (yellow) and human (blue) are colored.

Study of the human CD8αα-p/MHC I complex showed that three residues, Ser34^hCD8α^, Tyr51^hCD8α^ and Asn99^hCD8α^ form hydrogen bonds with the side chains of Gln226, Asp227 and Leu230 of CD loop and D strand of human MHC I α3 domain (16). Notably, the side chain of Gln226 from MHC I protrudes into the cavity of CD8α where it forms a bond with Ser34 in the C strand of CD8α (**Figure 8A**). This cavity and the key residues for interaction with MHC I are highly conserved in chickens and mammals (18, 31). While Tyr51^hCD8α^ and Asn99^hCD8α^ are conserved in cartilaginous fish CD8αs, a His residue is found in the position corresponding to Ser32^hCD8α^ in *Sc*CD8αα (**Figure 3**). Correspondingly, the Gln residue of MHC I that interacts with Ser32^hCD8α^ in endothermic species is replaced by a negatively charged Glu in almost all ectotherms, potentially strengthening the interaction through formation of an ionic bond.

The variation and a shift in the spatial location of the key CD loop residues in the α3 domain of MHC I plays a critical role in maintaining the interaction with CD8. In human MHC I (PDB code: 1AKJ), the key amino acid in the CD loop is Gln226, whereas in MHC I of nurse shark (PDB code:6LUP), grass carp (PDB code:5Y91), African clawed frog (*Xenopus laevis*) (PDB code: 6A2B), Green Anole Lizard (PDB code:7CPO) and chicken (PDB code: 4E0R), it is Asp220, Glu219, His224, Gln226 and Gln222 respectively. Superposition of representative MHC I and CD8αα structures, as shown in **Figure 8B**, taking Gln226 of human MHC-I as a reference, show the distance from Gln226 to Asp220, Glu219, His224, Gln226 and Gln222 is ~9.0 Å, ~13.2 Å, ~8.6 Å, ~7.6 Å and ~7.4 Å respectively. Thus, distance between CD8αα and p/MHC I decreases from lower to higher vertebrates (**Figure 8B**). Grass carp CD8αα, of which the residue Ser32^hCD8α^ is replaced by Phe33, still can interact with MHC I. Notably, residue Gln is responsible to interact with Ser in MHC I of most endotherm, while it is replaced by a negative charged Glu in almost all MHC I of ectotherm. Together, our data indicate that CD8αα is capable of interaction with MHC I and that the interaction between MHC I and CD8α differs between mammalian and non-mammalian species.

## Discussion

The cartilaginous fishes, including sharks, are an ancient lineage which diverged from other vertebrates approx. 450 million years ago (2, 32). In addition to Igs, cartilaginous fishes possess a complete CD8/MHC I system. Recently, the structure of nurse shark MHC I was solved and showed many features, including the interactions between the MHC I heavy chain, β2-microglobulin (β2m) and peptide ligand (21), are similar to those of bony vertebrates. To investigate the interaction between CD8 and MHC I in cartilaginous fishes, *CD8α* and *CD8β* were cloned from small-spotted catshark and the structure of *Sc*CD8αα solved. This is the first reported CD8αα structure from a cartilaginous fish.

Our gene synteny analysis shows that the *CD8α* and *CD8β* genes are tandemly linked from cartilaginous fishes to humans (31, 33), suggesting the duplication giving rise to these genes occurred in an ancestor of all jawed vertebrates. Despite low sequence identity (25.8%) between *Sc*CD8α and *Sc*CD8*β*, their IgSF V extracellular domains, which are vital for their interaction with MHC I (20, 34), share a similar domain architecture. However, the topology of *Sc*CD8α displays significant differences with CD8αs previously characterized in endotherms. Most notably, the A strand of *Sc*CD8α is located in the rear β sheet (**Figure 2**) but in the front sheet of chicken and human CD8α (16, 18), however, it should be noted that A strand exhibits considerable variations in all known structures of CD8α (17, 19). Further, an A’ strand is present only in the CD8α structures of catshark, grass carp and swine (17, 19). Therefore, we propose that the ancestral CD8α molecule likely adopted the topological conformation comprising A and A’ strands.

Our study also demonstrated that, as in mammals (16), cartilaginous fish CD8α can form a homodimer (**Supplementary Figure 3**). Our PAGE analysis under non-reducing conditions clearly showed a single band of approx. 32 kDa, matching the size of *Sc*CD8α homodimer (32.1 kDa). Previous analyses have shown that a hydrophobic core, predominantly involving hydrophobic aromatic residues, serves as the key interaction force which maintains CD8α dimerization (19). A similar hydrophobic core can be found in the *Sc*CD8αα structure (**Figure 6**). Moreover, the aromatic residues forming the hydrophobic core of *Sc*CD8αα, including Phe30*^Sc^* ^CD8α^, Phe40*^Sc^* ^CD8α^, Tyr43*^Sc^* ^CD8α^, Tyr83*^Sc^* ^CD8α^ and Phe94*^Sc^* ^CD8α^, are relatively well conserved in vertebrates (**Figure 5**). This suggests the mechanism of CD8αα-MHC I interaction is evolutionarily conserved from cartilaginous fishes through to mammals.

So far structures of the CD8αα-p/MHC I complex have been solved for humans, mice and chickens (16, 18, 31). In all these species the cavity formed by the CDR loops of CD8αα is the principal area of interaction with p/MHC I (16, 18, 31). In humans, Ser34, Tyr51 and Asn99 of CD8α are vital for connection with the side chains of Gln226, Asp227 and Leu230 of MHC I (16). Ma et al. (35) report that the distance between CD8α and pMHC I within the interface decreased from Xenopus to mammals (35), which is believed to contribute to the stronger interactions seen in mammals. The structure of nurse shark MHC I was recently solved (28). Structural modeling revealed that the CD loop in the α3 domain of nurse shark MHC I is protruded ~9.0 Å shorter in the cavity relative to that of human MHC I (**Figure 8**), suggesting that the distance between shark CD8α and pMHC I may be longer than that in humans and as a result, the interaction may be weaker.

The structure of *Sc*CD8αα shows a canonical cavity is formed between the CDR loops of the two monomers. This suggests that the primordial CD8αα homodimer possesses the core structural unit to interact with p/MHC I. This cavity has been found in all known CD8αα structures except grass carp CD8αα, where the cavity is notably absent (16–19). Despite lack of the cavity, grass carp CD8αα can still bind to p/UAA-β2m (19). While this suggests that formation of the cavity is not essential for the interaction between CD8α and p/MHC I-β2m and that the interaction may be different in grass carp/bony fish relative to other vertebrates.

In summary, this study identified the *CD8α* and *CD8β* genes in small-spotted catshark and solved the structure of CD8αα. The *ScCD8α* and *ScCD8β* genes are found to be tandemly clustered in the genome and their encoded proteins share a characteristic IgSF V domain in their extracellular region. It was shown that *Sc*CD8α can form a homodimer or a heterodimer with *Sc*CD8β. Our structure revealed that *Sc*CD8αα, like its mammalian counterparts, mainly relies upon a hydrophobic core to form a dimeric structure. Furthermore, *Sc*CD8αα has a canonical cavity which mediates the interaction with p/MHC I in most species examined.

## Data availability statement

The datasets presented in this study can be found in online repositories. The names of the repository/repositories and accession number(s) can be found in the article/**Supplementary Material**.

## Ethics statement

The animal study was reviewed and approved by University of Aberdeen’s Animal Welfare and Ethical Review Body (AWERB).

## Author contributions

ZJ and JF: investigation, methodology, data curation, and writing original draft. HD: conceptualization, supervision and editing. JZ: conceptualization, funding acquisition, project administration, supervision and editing. JW: conceptualization, data curation, project administration, supervision and editing. All authors contributed to the article and approved the submitted version.
