# Supplementary material for: The first crystal structure of CD8αα from a cartilaginous fish
Source: Front Immunol. 2023 Apr 14;14:1156219. doi: 10.3389/fimmu.2023.1156219 (PMC10140343; doi:10.3389/fimmu.2023.1156219)
Supplement: Supplementary file 1 [file DataSheet_1.docx]

Supplementary Figure 1


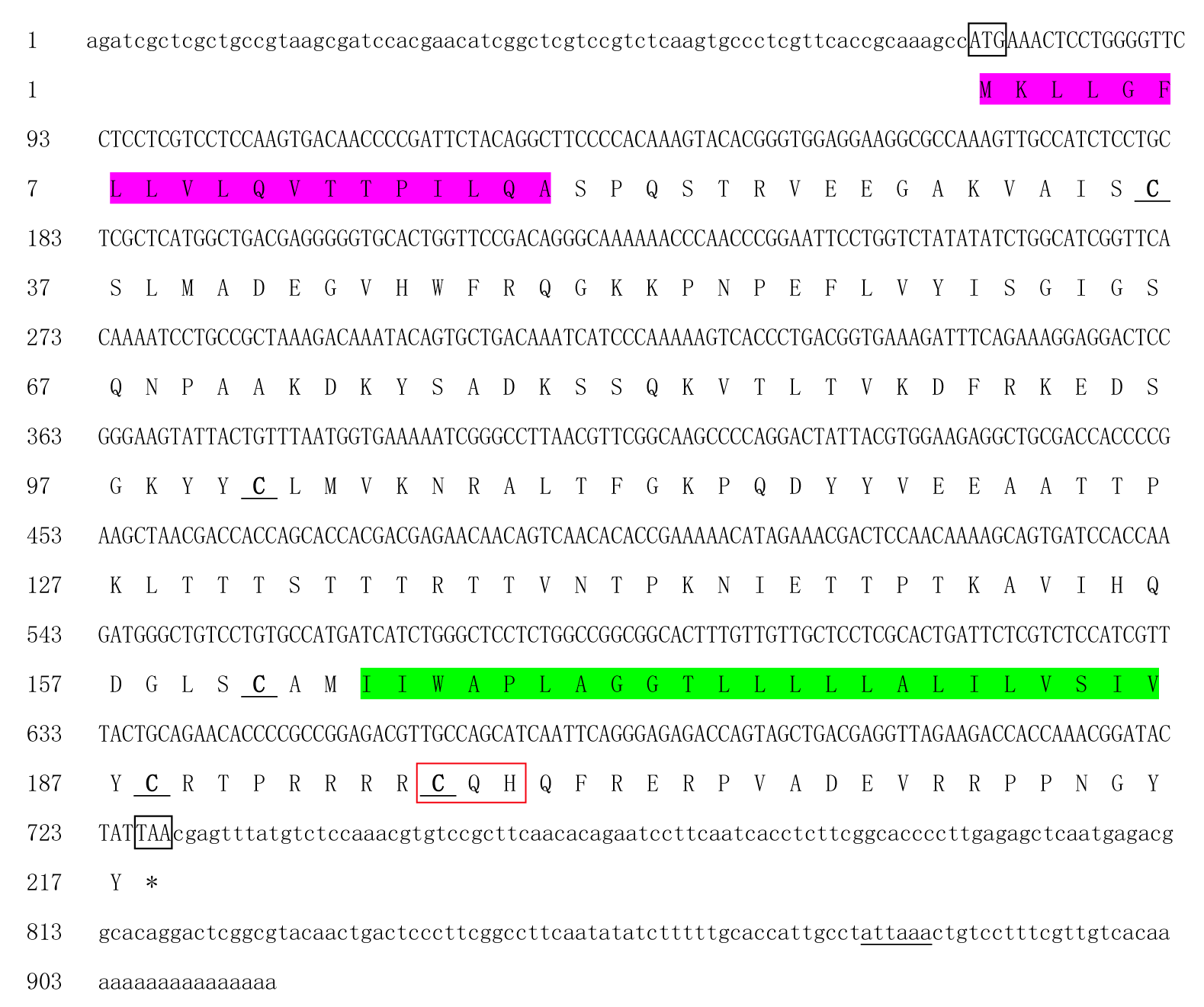


Supplementary Figure 1. Nucleotide and deduced amino acid sequences of *Sc*CD8α. Predicted signal peptide (magenta) and transmembrane region (green) are highlighted. Black boxed sequences are the protein translation start and stop codon. Red boxed is the CXH motif. The cysteine residues and the polyadenylation signal motif (attaaa) motif are underlined.

Supplementary Figure 2


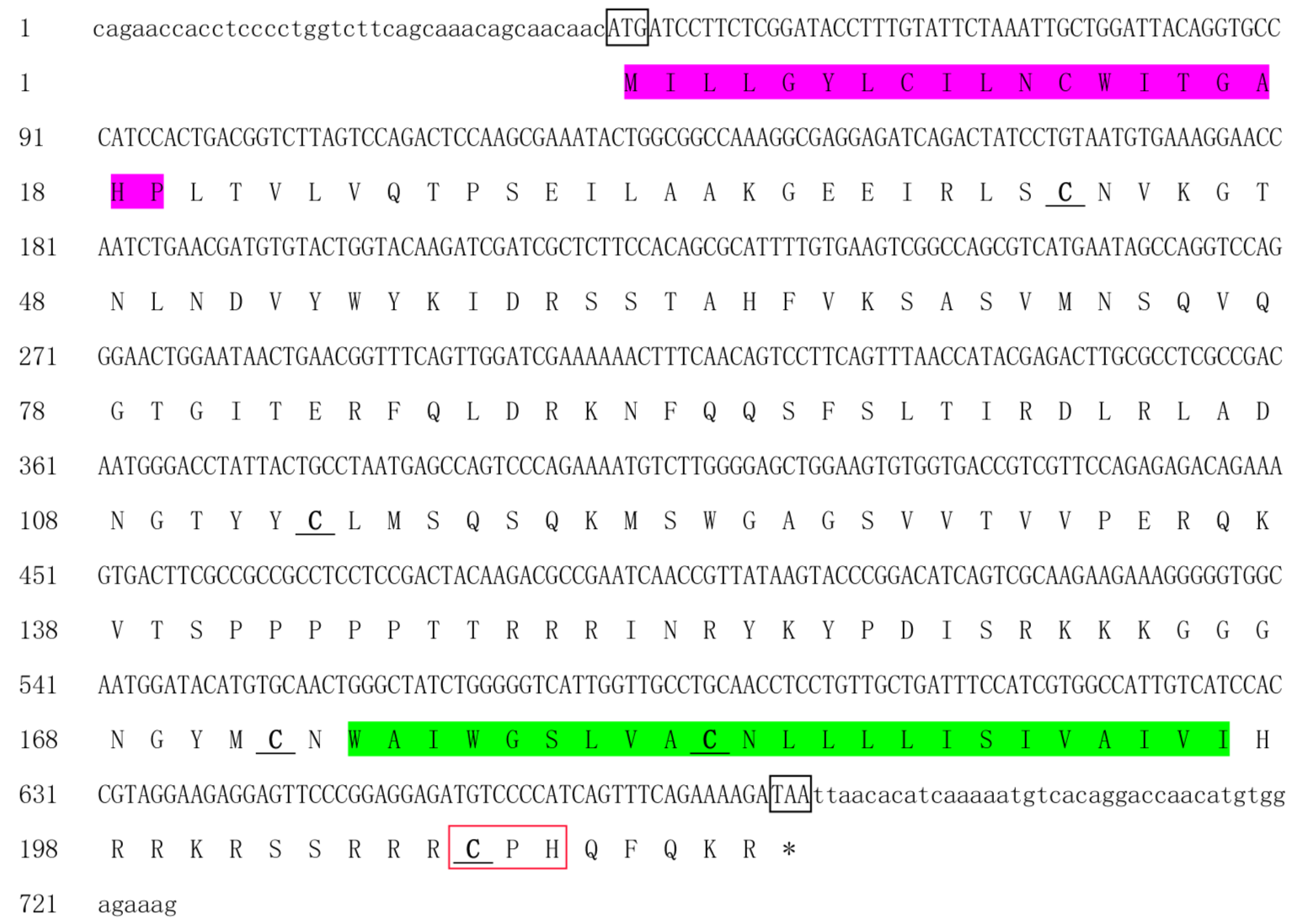


Supplementary Figure 2. Nucleotide and deduced amino acid sequences of *Sc*CD8β. Predicted signal peptide (magenta) and transmembrane region (green) are highlighted. Black boxed sequences are the protein translation start and stop codon. Red boxed is the CXH motif. The cysteine residues are underlined.

Supplementary Figure 3


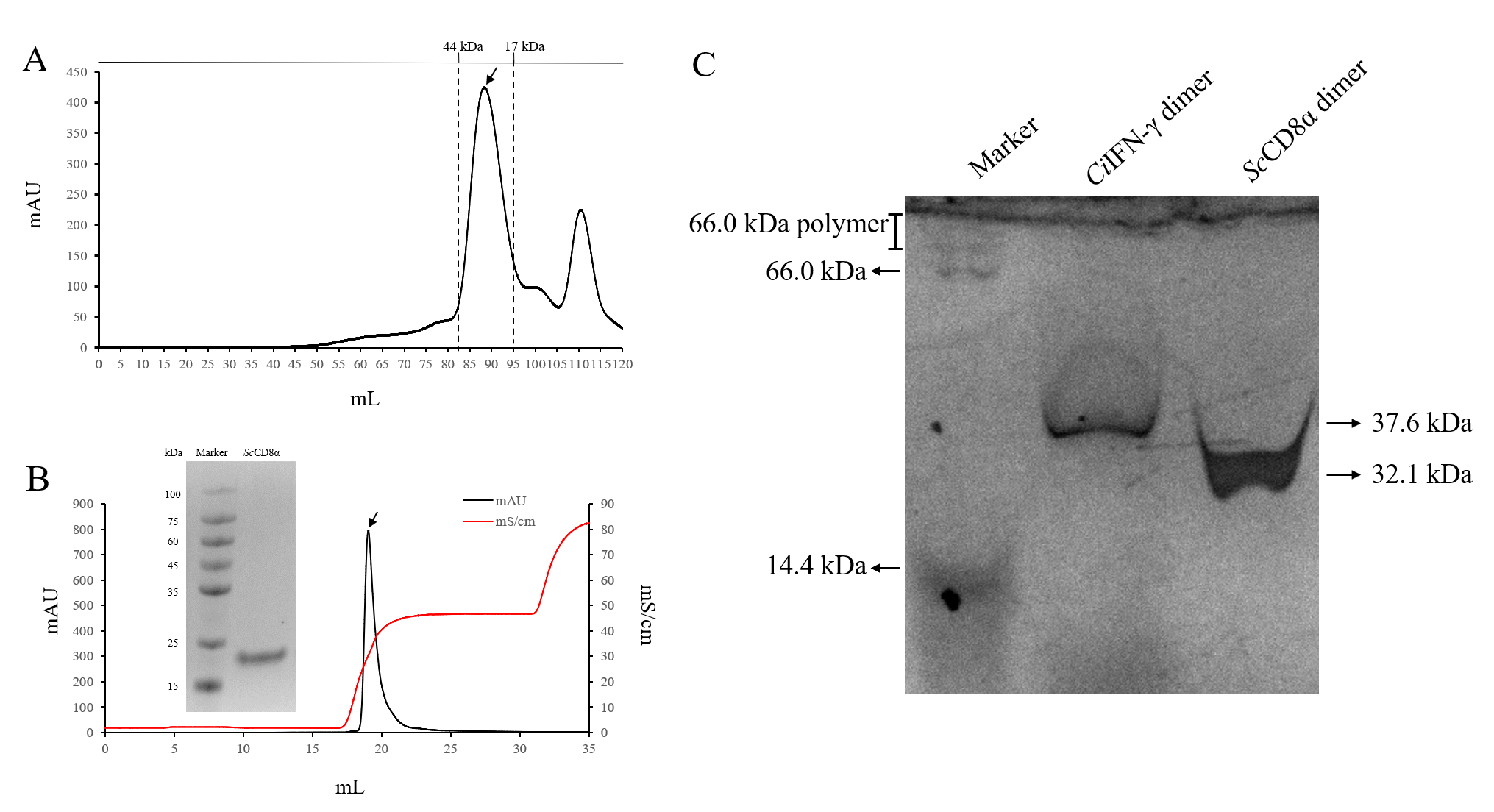


Supplementary Figure 3**.** (**A**) Purification of recombinant *Sc*CD8α protein using a Superdex 200 16/600 column. The molecular size of the eluted *Sc*CD8α (indicated by arrow) was estimated to be between 17 kDa (95 ml) and 44 kDa (82 ml) based on the protein reference for the column. (**B**) Purification of recombinant *Sc*CD8α protein by Resource Q anion-exchange chromatography and SDS-PAGE analysis under reducing conditions. (**C**) PAGE analysis of recombinant *Sc*CD8α protein under non-reducing conditions.

Supplementary Figure 4


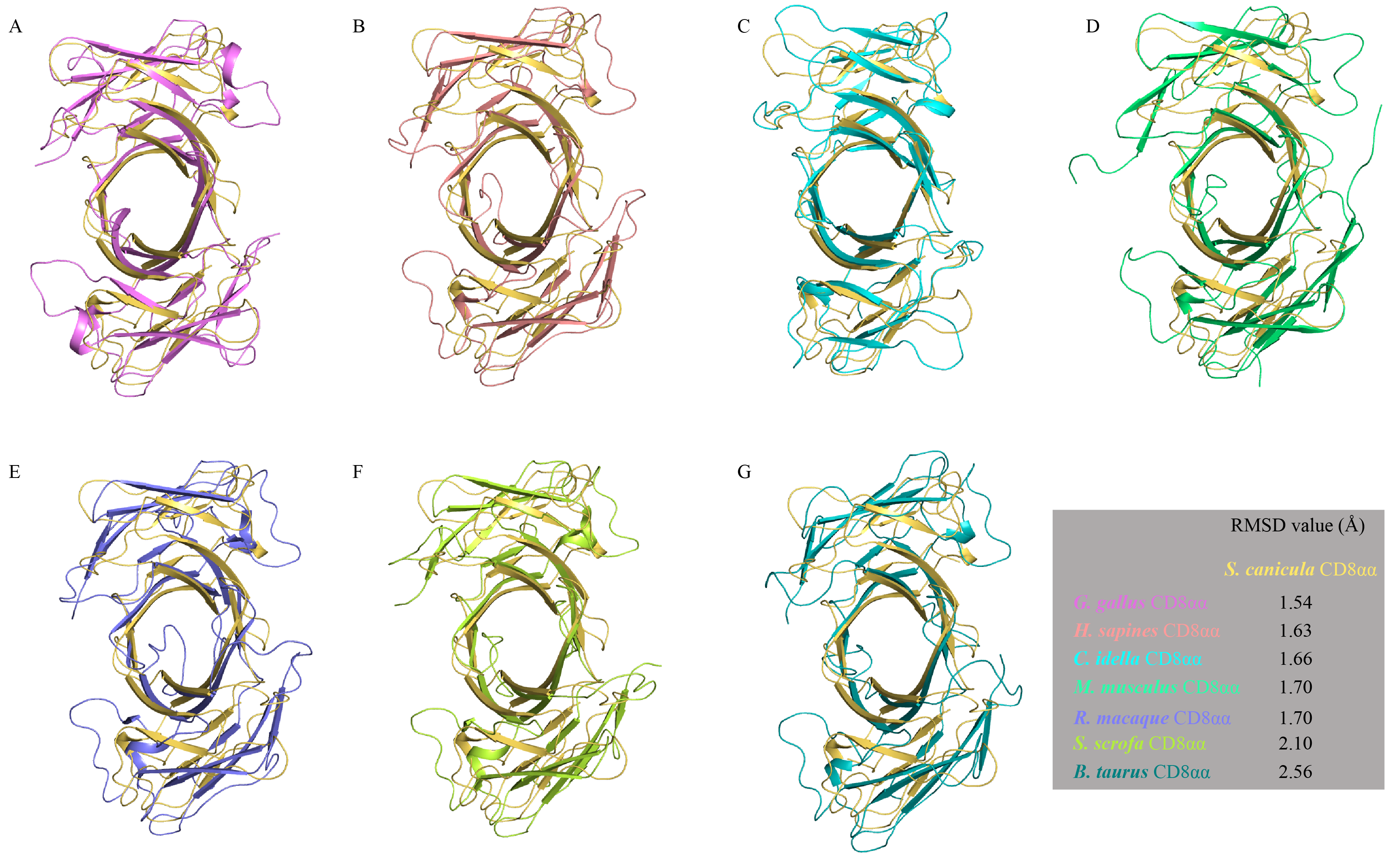


Supplementary Figure 4. Structural alignment of *Sc*CD8α with known CD8αs. The *Sc*CD8α was superposed with *G. gallus* (**A**), *H. sapiens* (**B**), *C. idella* (**C**), *M. musculus* (**D**), *R. macaque* (**E**), *S. scrofa* (**F**) and *B. taurus* (**G**). The RMSD values are shown. The CD8α structural data of *G. gallus* (5EB9), *H. sapines* (1CD8), *C. idella* (5Z11), *M. musculus* (2ARJ), *R. macaque* (2Q3A), *S. scrofa* (5EDX) and *B. taurus* (5EBG) were retrieved from the PDB database.

**Supplementary Table 1** Information of sequences for bioinformatics analysis

| Gene | Species | Accession |
| --- | --- | --- |
| CD8α | *Ginglymostoma cirratum* | AGN91183.1 |
|  | *Rhincodon typus* | XP_020369957.1 |
|  | *Rhinobatos productus* | ABQ85060.1 |
|  | *Scyliorhinus canicula* | MT840192 |
|  | *Xenopus tropicalis* | XP_031750940.1 |
|  | *Xenopus laevis* | ADV71260.1 |
|  | *Alligator mississippiensis* | XP_014463121 |
|  | *Chrysemys picta bellii* | XP_005315058 |
|  | *Oxyura jamaicensis* | XP_035182000.1 |
|  | *Gallus gallus* | NP_990566 |
|  | *Mus musculus* | NP_001074579 |
|  | *Sus scrofa* | NP_001001907 |
|  | *Bos taurus* | XP_005900430 |
|  | *Macaca mulatta* | XP_001092778 |
|  | *Homo sapiens* | NP_001139345 |
|  | *Ctenopharyngodon idella* | ACU30711.1 |
|  | *Danio rerio* | NP_001035138.1 |
|  | *Dicentrarchus labrax* | XP_051269742.1 |
|  | *Oncorhynchus kisutch* | XP_020337852.1 |
|  | *Salmo salar* | NP_001117055.1 |
|  | *Microcaecilia unicolor* | XP_030046904.1 |
| CD8β | *Scyliorhinus torazame* | GCB70529.1 |
|  | *Scyliorhinus canicula* | MW713126.1 |
|  | *Ginglymostoma cirratum* | AGQ17914.1 |
|  | *Xenopus laevis* | ADV71261.1 |
|  | *Xenopus tropicalis* | XP_031750941.1 |
|  | *Alligator mississippiensis* | XP_019333895.1 |
|  | *Chrysemys picta bellii* | XP_023962252.2 |
|  | *Oxyura jamaicensis* | XP_035182001.1 |
|  | *Gallus gallus* | NP_990578.3 |
|  | *Mus musculus* | NP_033988.1 |
|  | *Sus scrofa* | NP_001335699.1 |
|  | *Bos taurus* | NP_001098814.1 |
|  | *Macaca mulatta* | XP_014968292.1 |
|  | *Homo sapiens* | NP_001171571.1 |
|  | *Ctenopharyngodon idella* | ACU30712.1 |
|  | *Danio rerio* | XP_001345248.4 |
|  | *Dicentrarchus labrax* | XP_051271588.1 |
|  | *Salmo salar* | XP_014054648.1 |
|  | *Oncorhynchus mykiss* | AAT68458.1 |
|  | *Ambystoma mexicanum* | AAF61253.1 |

**Supplementary Table 2** Percentages of sequence identity between CD8αs and CD8βs.

| Species | *Sc*CD8α | *Sc*CD8β | *Hs*CD8α | *Hs*CD8β | *Mm*CD8α | *Mm*CD8β | *Gg*CD8α | *Dr*CD8α | *Dr*CD8β |
| --- | --- | --- | --- | --- | --- | --- | --- | --- | --- |
| *Sc*CD8α |  |  |  |  |  |  |  |  |  |
| *Sc*CD8β | 25.8 |  |  |  |  |  |  |  |  |
| *Hs*CD8α | 25.9 | 22.8 |  |  |  |  |  |  |  |
| *Hs*CD8β | 26.9 | 26.4 | 22.5 |  |  |  |  |  |  |
| *Mm*CD8α | 28.1 | 21.4 | 49.6 | 22.0 |  |  |  |  |  |
| *Mm*CD8β | 23.4 | 24.6 | 18.1 | 52.3 | 20.7 |  |  |  |  |
| *Gg*CD8α | 29.2 | 20.3 | 33.5 | 24.5 | 29.3 | 17.9 |  |  |  |
| *Gg*CD8β | 26.1 | 28.8 | 24.3 | 33.8 | 24.5 | 28.8 | 24.5 |  |  |
| *Dr*CD8α | 25.9 | 20.2 | 20.8 | 21.1 | 22.1 | 22.2 | 22.7 | 20.2 |  |
| *Dr*CD8β | 22.8 | 22.3 | 20.8 | 22.6 | 21.2 | 23.2 | 17.4 | 26.5 | 23.0 |

*Dr, Danio rerio; Gg, Gallus gallus; Hs: Homo sapiens; Mm, Mus musculus; Sc, Scyliorhinus canicular.*
